# Multidrug-Resistant Pandemic (H1N1) 2009 Infection in Immunocompetent Child

**DOI:** 10.3201/eid1708.102004

**Published:** 2011-08

**Authors:** AliReza Eshaghi, Samir N. Patel, Alicia Sarabia, Rachel R. Higgins, Alexei Savchenko, Peter J. Stojios, Yan Li, Nathalie Bastien, David C. Alexander, Donald E. Low, Jonathan B. Gubbay

**Affiliations:** Author affiliations: Ontario Agency for Health Protection and Promotion, Toronto, Ontario, Canada (A. Eshaghi, S.N. Patel, R.R. Higgins, D.C. Alexander, D.E. Low, J.B. Gubbay);; The Credit Valley Hospital, Mississauga, Ontario, Canada (A. Sarabia);; University of Toronto, Toronto (A. Savchenko, P.J. Stojios, D.C. Alexander, D.E. Low, J.B. Gubbay);; Public Health Agency of Canada, Winnipeg, Manitoba, Canada (Y. Li, N. Bastien);; Mount Sinai Hospital, Toronto (D.E. Low, J.B. Gubbay);; The Hospital for Sick Children, Toronto (J.B. Gubbay)

**Keywords:** viruses, influenza A, H1N1, pandemic, drug resistant, oseltamivir, zanamivir, dispatch, expedited

## Abstract

Recent case reports describe multidrug-resistant influenza A pandemic (H1N1) 2009 virus infection in immunocompromised patients exposed to neuraminidase inhibitors because of an I223R neuraminidase mutation. We report a case of multidrug-resistant pandemic (H1N1) 2009 bearing the I223R mutation in an ambulatory child with no previous exposure to neuraminidase inhibitors.

The neuraminidase inhibitors (NAIs) oseltamivir and zanamivir are antiviral agents approved for treatment of infections caused by pandemic (H1N1) 2009 influenza virus. Since the 2008–09 influenza season, almost all seasonal influenza (H1N1) viruses have been oseltamivir resistant because of an H275Y (histidine to tyrosine NA mutation, N1 NA numbering) mutation. Despite widespread use of oseltamivir during the 2009 pandemic, NAI resistance is rare in pandemic (H1N1) 2009 viruses (*1*). Zanamivir resistance is also rare in influenza viruses. A Q136K (glutamine to lysine mutation, N2 NA numbering) mutation conferring zanamivir resistance in influenza (H1N1) viruses has been described in an in vitro study but has not been detected in clinical specimens from patients (*2*). An influenza B strain carrying a R152K (arginine to lysine) mutation and resistant to oseltamivir and zanamivir has been reported (*3*). Recent case reports described multidrug-resistant pandemic (H1N1) 2009 infection in immunocompromised patients exposed to oseltamivir and zanamivir because of an I223R (isoleucine to arginine) mutation in NA (*4**–**6*). We report a case of infection by multidrug-resistant pandemic (H1N1) 2009 virus bearing the I223R mutation in an ambulatory child with no previous exposure to NAI.

## The Study

On October 30, 2009, a 15-year-old girl with a history of asthma sought treatment at an emergency department in the Greater Toronto area after 3 days of cough and rhinorrhea and 1 day of chest pain. Several children at her school also had respiratory symptoms. On arrival, she was febrile to 39.6°C and mildly dehydrated; physical examination was otherwise unremarkable. Blood count and chest radiograph showed no abnormalities. The child received intravenous rehydration in the emergency department, was discharged home with a prescription for oseltamivir therapy, and recovered uneventfully. A nasopharyngeal swab was forwarded to Ontario Agency for Health Protection and Promotion (OAHPP) for influenza testing. Pandemic (H1N1) 2009 was detected by real-time reverse transcription PCR (*7*). Subsequently, the specimen was screened by a single-nucleotide polymorphism assay distributed by Canada’s National Microbiology Laboratory and the World Health Organization pyrosequencing protocol for the presence of the H275Y mutation (*8*). Both assays confirmed the isolate was wild type (histidine) at aa 275 of NA.

As part of pandemic surveillance, the specimen was cultured in rhesus monkey kidney cells and whole genome sequencing was performed by using a modified World Health Organization protocol (*9*). Sequences were deposited into GenBank under accession nos. CY060619–CY060626. In comparison with A/California/7/2009 (H1N1), several nonsynonymous mutations were identified: I201V and E538K in polymerase; S220T, D239E, and K465R in hemagglutinin; V100I and M316I in nucleoprotein; S99P and I123V in nonstructural protein; T16I, V106I, I223R, N248D, and N369K in NA. Apart from I201V, which is of unknown significance and has not been previously documented in pandemic (H1N1) 2009, these mutations were detected in 22% to 72% of pandemic (H1N1) 2009 strains circulating in Ontario at the same time that underwent whole genome sequencing. The I223R mutation results from a 1 nucleotide substitution at codon 223 of NA. To rule out the possibility of acquisition of I223R during culture in rhesus monkey kidney cells, the NA gene of the primary sample and its first passage were sequenced. Both had 100% identical nucleotide composition.

The 50% inhibitory concentration (IC_50_) values for oseltamivir carboxylate and zanamivir, determined by chemiluminescent NAI assay (NA-Star; Applied Biosystems Ltd., Foster City, California, USA) at OAHPP, were 9.49 (SD ± 2.19) nmol and 2.46 (SD ± 0.30) nmol, respectively (Table 1, Table 2) (oseltamivir carboxylate and zanamivir supplied by Hoffmann-La Roche Ltd [Basel, Switzerland] and GlaxoSmithKline [Brentford, UK], respectively). Compared with a wild-type control, the I223R mutant exhibited 28- and 12-fold increases in IC_50_s for oseltamivir and zanamivir, respectively. The oseltamivir IC_50_ of the I223R strain was elevated, but not as much as observed in an H275Y control, which had a 168-fold IC_50_ elevation compared to the wild-type strain and was 6× higher than that of the I223R strain when tested in parallel. Similar results were obtained when the sample was retested at the National Microbiology Laboratory (Table 1, Table 2).

**Table 1:** Susceptibility of I223R mutant and control pandemic (H1N1) 2009 strains to oseltamivir carboxylate in the chemiluminescent NA inhibition assay, Canada, 2010*

Virus strain	NA mutation†	Susceptibility				
OAHPP testing			NML testing	
Mean IC_50_ ± SD, nmol	-fold increase		Mean IC_50_ ± SD, nmol	-fold increase
A/Ontario/313762/2009	I223R	9.49 ± 2.19‡	28		10.95 ± 2.5‡	22
A/California/07/2009-like control	Wild type	0.34 ± 0.14§			0.49 ± 0.31§	
Oseltamivir-resistant control	H275Y	57.1 ± 21.48¶	168		81.42 ± 24.1¶	166

*NA, neuraminidase; OAHPP, Ontario Agency for Health Protection and Promotion; NML, National Microbiology Laboratory; IC_50_, 50% inhibitory concentration. †Mutations presented in N1 numbering. ‡For 7 and 4 experiments done by OAHPP and NML, respectively. §For 17 and 1,446 experiments done by OAHPP and NML, respectively. ¶For 13 and 14 experiments done by OAHPP and NML, respectively.

**Table 2:** Susceptibility of I223R mutant and control pandemic (H1N1) 2009 strains to zanamivir in the chemiluminescent NA inhibition assay, 2010*

Virus strain	NA mutation	Susceptibility				
OAHPP testing			NML testing	
Mean IC_50_ ± SD, nmol	-fold increase		Mean IC_50_ ± SD, nmol	-fold increase
A/Ontario/313762/2009	I223R	2.46 ± 0.30†	12		6.84 ± 1.3†	9
A/California/07/2009-like control	Wild type	0.20 ± 0.11‡			0.79 ± 0.45‡	
Oseltamivir-resistant -control	H275Y	0.20 ± 0.05§			0.76 ± 0.35§	

*NA, neuraminidase; OAHPP, Ontario Agency for Health Protection and Promotion; NML, National Microbiology Laboratoy; IC_50_, 50% inhibitory concentration. †For 7 and 4 experiments done by OAHPP and NML, respectively. ‡For 15 and 1,446 experiments done by OAHPP and NML, respectively. §For 9 and 14 experiments done by OAHPP and NML, respectively.

The clinical significance of the I223R mutation is poorly understood because the IC_50_s for oseltamivir and zanamivir are well below achievable serum levels when administered at recommended doses. Oral oseltamivir at a dose of 75 mg 2×/d resulted in a maximum serum concentration (C_max_) of 348 ng/mL (1,115 nmol). Repeated inhalation of 10 mg of the dried powder formulation of zanamivir produced a C_max_ of 39 to 54 ng/mL (117.5–162.7 nmol) at 1 to 2 h postdose, with an elimination half life of 4–5 h (*10*). Intravenous zanamivir at a dose of 600mg resulted in a C_max_ of 32,000–39,000 ng/mL (96,300–117,360 nmol).

I223 is recognized as one of the framework residues responsible for stabilizing the NAI active site; type-specific mutations at these residues have resulted in reduced susceptibility to NAIs (*11**,**12*). Although the exact mechanism by which mutations at the framework residue alter susceptibility to particular NAIs is not clear, simulation studies suggest that the NA electrostatic potential plays a major role in the interaction and stabilization of NAIs within the NA cavity (*13*). Nonhomologous substitution of a nonpolar hydrophobic amino acid, isoleucine, with the positively charged (polar) hydrophilic amino acid, arginine (I223R), seems to be a key point in alteration of the NA cavity. These changes most likely result in active site endpoint interactions affecting drug binding affinity and could disturb the proposed electrostatic binding funnel instrumental in directing NAIs into and out of binding sites on NA (*14*).

Three independent case reports described infections caused by multidrug-resistant pandemic (H1N1) 2009 in immunocompromised patients who received prolonged treatment with oseltamivir followed by zanamivir; 2 of the infections were fatal. In 2 patients, infection developed (H275Y followed by I223R alone with simultaneous reversion to wild type at position 275) (*4**,**5*); dual H275Y/I223R mutations developed in the third patient (*6*). Our patient is unique because she was immunocompetent, had no prior exposure to NAIs, and had an uneventful recovery. A similar resistance profile was seen in the published case exhibiting I223R alone, where IC_50_s for oseltamivir, zanamivir, and peramivir were elevated by 45-, 10-, and 7-fold, respectively (*4*). The origin of the multiresistant isolate in this patient’s case could not be established. The I223R mutation may have occurred spontaneously in our patient. Alternatively, she acquired infection in the ambulatory setting, possibly as part of a school outbreak. Resistance may have evolved following random mutation, or during NAI therapy in another patient. We could not investigate this further because no samples were submitted from contacts. Using reverse genetics, it has been recently shown that an I223V NA change increased oseltamivir and peramivir resistance in pandemic (H1N1) 2009 and also restored NA substrate affinity and replication fitness in vitro (*15*).

## Conclusions

Although the I223 residue is highly conserved across pandemic (H1N1) 2009 strains, the global distribution of pandemic (H1N1) 2009 was made possible by the virus adapting for stable circulation through genetic changes contributing to fitness and facilitating transmissibility from person to person. This report of community acquisition of a multidrug-resistant strain of pandemic (H1N1) 2009 reinforces the need to continue close monitoring for the emergence of resistant viruses and incorporation of screening for newly discovered resistance mutations into clinical diagnostics.
